# Dysregulation and chronicity of pathogenic T cell responses in the pre-diseased stage of lupus

**DOI:** 10.3389/fimmu.2022.1007078

**Published:** 2022-10-28

**Authors:** Justus Ohmes, Sara Comdühr, Reza Akbarzadeh, Gabriela Riemekasten, Jens Y. Humrich

**Affiliations:** Department of Rheumatology and Clinical Immunology, University of Lübeck, Lübeck, Germany

**Keywords:** systemic lupus erythematosus, autoimmunity, immune regulation, T cell signaling, metabolism, genetics, IL-2

## Abstract

In the normal immune system, T cell activation is tightly regulated and controlled at several levels to ensure that activation occurs in the right context to prevent the development of pathologic conditions such as autoimmunity or other harmful immune responses. CD4^+^FoxP3^+^ regulatory T cells (Treg) are crucial for the regulation of T cell responses in the peripheral lymphatic organs and thus for the prevention and control of autoimmunity. In systemic lupus erythematosus (SLE), a prototypic systemic autoimmune disease with complex etiology, a disbalance between Treg and pathogenic effector/memory CD4^+^ T cells develops during disease progression indicating that gradual loss of control over T cell activation is an important event in the immune pathogenesis. This progressive failure to adequately regulate the activation of autoreactive T cells facilitates chronic activation and effector/memory differentiation of pathogenic T cells, which are considered to contribute significantly to the induction and perpetuation of autoimmune processes and tissue inflammation in SLE. However, in particular in humans, little is known about the factors which drive the escape from immune regulation and the chronicity of pathogenic T cell responses in an early stage of autoimmune disease when clinical symptoms are still unapparent. Here we briefly summarize important findings and discuss current views and models on the mechanisms related to the dysregulation of T cell responses which promotes chronicity and pathogenic memory differentiation with a focus on the early stage of disease in lupus-prone individuals.

## Introduction

Systemic lupus erythematosus (SLE) is a severe multisystem autoimmune disease with complex pathogenesis which is characterized by immune dysregulation and chronic inflammation of various organs caused by the breach of immune tolerance predominantly towards nuclear autoantigens, such as double-stranded deoxyribonucleic acid (dsDNA). SLE primarily affects young women of childbearing age and clinical manifestations can range from relatively mild skin and joint involvement to life-threatening disease including renal, neurologic or cardiac inflammation (1, 2). Tissue inflammation and damage are mediated by the deposition of immune complexes and by the infiltration with autoreactive lymphocytes (2, 3). In particular, autoreactive CD4^+^ T cells are considered to play a central role in the initiation and perpetuation of the pathologic immune responses in several ways: Follicular T helper (Tfh) cells, which represent a subset of CD4^+^ T helper cells and which have the unique capability to migrate to the outer edge of the B cell follicles in the lymphatic organs in order to initiate germinal-center reactions (4), are essential for the activation and differentiation of autoreactive B cells in SLE (5, 6). Further, CD4^+^ effector T cells invade the affected tissues and mediate tissue inflammation and damage by cell-cell interactions and by the production of inflammatory and cytotoxic cytokines such as interferon (IFN)-γ and IL-17 (6, 7). In addition, chronic activation of CD4^+^ T cells and the generation of a robust autoimmune T cell memory may contribute to the recurrence of disease flares, the persistence of tissue inflammation and damage accrual and to treatment refractory disease states (6, 8). Despite the controlled deletion of most autoreactive T cells during T cell maturation in the thymus, T cells that can recognize autoantigens are still abundantly present in the healthy organism, but those do not become pathogenic when kept under check by intact mechanisms of peripheral self-tolerance (9). Regulatory CD4^+^ T cells (Treg) expressing the linage specific transcription factor forkhead box P3 (FoxP3) are indispensable for the maintenance of peripheral self-tolerance and thus for the prevention and control of inflammation and autoimmunity throughout entire life. Predominantly derived from a predetermined T cell subpopulation in the thymus, CD4^+^FoxP3^+^ Treg mainly recognize auto-antigens *via* their T cell receptor and are required to regulate the activation and expansion of auto-reactive T cells and other harmful immune cells in the peripheral lymphatic organs (10–13). Given their crucial function in immunoregulation and peripheral tolerance, it appears obvious that disturbances in Treg biology contribute to the development of autoimmune diseases such as SLE (14, 15). Indeed, it was shown in murine and later also in human SLE that an acquired and progressive deficiency of the cytokine interleukin-2 (IL-2), an essential growth and survival factor for Treg, promotes an imbalance between Treg and effector/memory CD4^+^ T cells, which was associated with accelerated disease activity (16–18). These pathophysiological findings have stimulated the successful translation of low-dose IL-2 therapy into clinical trials aiming to restore Treg activity in patients with active SLE (19–24).

Given the pivotal role of T cells and their dysregulation in SLE pathogenesis, it can be hypothesized that chronic T cell activation and memory differentiation in early disease are key events for the initiation and progression of autoimmunity in SLE.

## Genetic basis of aberrant T cell activation in human and murine SLE

The development and progression of SLE involves a complex interaction of genetic risk, diet, environmental influences, and immune dysregulation (3, 25). Individuals with genetic risk alleles for SLE who are exposed to environmental risk factors during their lifetime could be more susceptible to autoimmune diseases where synergistic interactions facilitate the onset of pathogenic autoimmune responses. Up to now, several lines of evidence indicate that genetic factors contribute to the etiopathogenesis of SLE, supported by twin studies or familial aggregation investigations (26). Specifically, GWAS data have uncovered the involvement of several susceptible HLA and non-HLA genes supporting that altered T cell signal transduction and activation is important in SLE. Various proteins such as cytokines and kinases essential for regulating T cell activation, proliferation and differentiation, are encoded by these susceptible loci. The HLA region contains several genes encoding for molecules involved in antigen presentation or immune-related proteins (27). In SLE, expression of HLA-DR, which is an indicator of activated T cells, is elevated in circulating T cells and the frequency of HLA-DR-expressing CD3^+^ T cells is associated with SLE disease severity (28). Besides HLA loci, genes outside the HLA region also appear to play an important role in SLE development. For instance, a mutation in the SLE risk gene *PTPN22* (R620W), encoding for a tyrosine phosphatase that regulates T cell signaling, is associated with aberrant receptor signaling function on effector and memory T cells as well as B cells (29). However, in polygenic diseases such as SLE genetic contribution could be distinctly involved in disease susceptibility at different ages (30) as suggested for pediatric and adult-onset SLE. Pediatric SLE patients, in particular those with monogenetic forms, are an inimitable group to highlight the importance of genetic contribution, as they develop disease earlier with a more severe disease manifestation and a higher frequency of family history (31). For example, a positive correlation between polymorphisms in HLA genes and the age of SLE diagnosis has been shown, indicating that older patients have the higher genetic risk (32). Conversely, higher number of SLE-associated non-HLA polymorphisms are prevalent in the younger patients (32). Analysis of candidate genes in children with SLE and their parents has confirmed the involvement of SLE-associated genes, including SELP (P-selectin gene) and IRAK1 (interleukin-1 receptor-associated kinase 1 gene), that are overexpressed in CD4^+^ Treg from patients with SLE (33, 34). Investigating both pediatric- and adult-onset patients with defined genetic defects could also provide valuable models to elucidate T cell dysregulation at the early phases of disease.

Parallel investigations over the last two decades indicated many similarities in the genetic basis for susceptibility to SLE between mice and men (3). In mice, the genetic involvement in SLE etiology is evidenced by various susceptible inbred mouse strains, which all develop a lupus-like disease, although to different extent, such as the (NZBxNZW) F1 (NZBW), NZM2410, and MRL–Fas^lpr^ strains (35). Similar to humans with SLE, GWAS has identified over 100 loci related to increased susceptibility for lupus-like disease in mice (36, 37). In the context of T cell-associated genetic alterations, the *Sle1a* gene segment in the NZM2410 lupus-prone strain is responsible for the increased activation of conventional CD4^+^ T cells (Tcon) and for the low numbers of CD4^+^FoxP3^+^ Treg (38, 39). The *Sle1c2* sublocus is another lupus susceptibility gene segment, which contributes to an elevated CD4^+^ T cell activation, a robust age-dependent expansion of IFN-γ-expressing Th1 cells, and a decrease in Treg counts (40). Signal transducer and activator of transcription (STAT) 4, a transcription factor engaged in the signal transduction of several cytokine receptors that plays a significant role in regulating T cell activation and differentiation is also a candidate gene for susceptibility to SLE. The deficiency of the *Stat4* gene in lupus-prone mouse models has confirmed its major effect on lupus severity, leading to reduced autoantibody production and T cell activation (41). Deficiency of *Fli1*, a transcription factor that is expressed by T cells, in MRL/lpr mice leads to diminished T cell activation, decreased expression of the Th1-associated chemokine receptor CXCR3 in T cells, and finally to reduced disease activity (42).

These findings in both humans and mice indicate that variants in several genes that are involved in T cell activation and differentiation are associated with SLE susceptibility and severity. In addition, CD4^+^ T cells from patients with active SLE exhibit a global DNA hypomethylation (43, 44) which is likely to cause an overexpression of numerous relevant genes.

## Abnormal T cell signaling in humans

Several studies have proven that T cells from SLE patients exhibit an abnormal signaling profile which can be detected already at the onset stage of disease (45, 46). One possible explanation for the excessive T cell response to antigen stimulation in SLE is related to an early abnormality in the molecular signaling pathway of T cells (3). In SLE patients, the complex of CD3 proteins, which is assembled with the TCR, shows defects in terms of a diminished expression of the CD3ζ chain, which is the only subunit that is both genetically and structurally distinct from the CD3δ, ϵ, and γ complex members (45, 47–49). In addition to the diminished expression of CD3ζ, it was shown that the functionally and structurally homologous Fc receptor gamma subunit (FcRγ) occupies the binding space of CD3ζ which may play a major role in the aberration of the antigen receptor-initiated signaling and therefore lead to a variety of pathogenic changes in the SLE T cell phenotype (46, 50). During the regular immune response in healthy individuals, CD3ζ recruits the tyrosine kinase zeta-chain-associated protein kinase-70 (ZAP-70), which ensures a controlled moderate calcium influx at the end of the signaling cascade (50, 51). In SLE T cells the replacement of CD3ζ with the FcRγ, induces the binding of the spleen tyrosine kinase (Syk) with high affinity instead of ZAP-70, which results in a much stronger calcium influx into the T cell cytoplasm and which in turn leads to a decreased activation threshold of CD4^+^ T and B cells upon autoantigen recognition. The increased intracellular calcium content leads to upregulation of calcium-triggered calcium/calmodulin-dependent protein kinase IV (CaMK4) (52, 53), which mainly regulates various transcription factors through phosphorylation, such as the cAMP-responsive element modulator α (CREMα) (54, 55). CREMα is known to negatively regulate IL-2 transcription and to induce the expression of IL-17 (56, 57), which is likely to promote the disbalance between Treg and Tcon, as the growth and survival of Treg are severely impaired due to the limited availability of IL-2.

The mammalian target of rapamycin (mTOR), a serine-threonine kinase localized in the outer mitochondrial membrane, has been identified as a central regulator of T cell lineage specification, serving as a physiological sensor of mitochondrial dysfunction and ATP depletion in T cells (58). mTOR translates a variety of environmental information into signals that control either nutrient supply, cAMP levels, and osmotic stress, as well as cellular processes including protein biosynthesis and autophagy (59). mTOR complex 1 (mTORC1) is considered essential for Th1 and Th17 differentiation, whereas mTOR complex 2 (mTORC2) is important for Th2 differentiation in mice (60). Both complexes suppress the transcription factor FoxP3 and thus inhibit the differentiation of FoxP3+ Treg (61). In SLE patients, it was shown that mTOR activation in double negative (DN) T cells was increased and preceded disease flares (62). In more detail, mTORC1 activity was found to be increased, whereas mTORC2 activity was reduced accompanied by an increase in Th17 cells (63). Consistent with this, inhibition of mTORC1 by rapamycin promoted the expansion of the CD4^+^FoxP3^+^ Treg population and the suppression of Th17 cells, and was capable to decrease disease activity in patients with active SLE (63–65).

Similarly, also the serine-threonine kinases Rho-associated protein kinases (ROCK) 1 and 2 play an important role in SLE pathogenesis. Generally, ROCKs regulate migration, activation, and differentiation of T cells and are crucial for controlling cytoskeletal components including the ezrin/radixin/moesin (ERM) proteins (45, 66). ERMs are important for the association of plasma membrane proteins with actin filaments, and regulate migration and cell adhesion through association with the intracellular domain of CD44 (67). Signaling through ROCK2 also plays an important role in the differentiation of Th17 cells and Tfh cells (45). PBMC and T cells from patients with SLE show significantly higher activity of ROCK and ERM compared to healthy controls (68, 69) and expression levels of CD44 are strongly increased in T cells and correlate with disease activity (68, 70), suggesting that increased adhesion and migration of SLE T cells occurs due to the steady activation of the CD44-ROCK-ERM axis (45).

Interferons (IFNs), in particular type I IFNs, play a central role as initiators of the pathogenic immune response in SLE. Nucleic acids released from apoptotic cells and immune complexes trigger the production type-1 IFNs by tissue resident plasmacytoid dendritic cells. Type-1 IFNs exert stimulatory effects on a variety of immune cells including T cells through activation of the STAT1 pathway (71–73). Consistent with this, it was reported that the expression levels of STAT1 were increased in CD4^+^ T cells from SLE patients and positively correlated with disease activity (74, 75). In addition, high levels of STAT1 phosphorylation were observed in activated Treg that were decreased in numbers, and it was shown that type-1 IFNs can induce apoptosis in Treg *via* the IRAK1 pathway (75, 76), indicating that type-1 IFNs negatively interfere with Treg homeostasis and survival.

Whether these abnormal signaling events are acquired during disease progression or genetically determined, however, remains to be determined.

## Abnormal T cell phenotype in humans

Up to now the earliest time point for which data on the T cell phenotype from SLE patients are available is at the onset of disease. In SLE patients with established clinical manifestations, different subsets of T cells with an abnormal activation pattern can be identified, which mediate inappropriate inflammatory responses and support enhanced B cell activation (3, 6). The frequencies of Ki67^+^, proliferating CD4^+^FoxP3^-^ conventional T cells (Tcon) is strongly increased in patients with active SLE and correlates with disease activity (17, 77), indicating that aberrant Tcon activation is associated with disease activity and severity. Th17 cells, a subset of CD4^+^ T helper cells, show an overactivation and express increased levels of IL-17, which promotes inflammation and systemic tissue damage by recruiting neutrophils, monocytes and other immune cells to the inflamed tissues and by inducing autoantibody production (78, 79). Interestingly, the rarely present double negative (DN) T lymphocytes lacking the CD4 and CD8 co-receptors (<5% of T lymphocytes in healthy individuals) are increased in SLE patients and induce the production of anti-dsDNA antibodies by autoreactive B cells. These DN T cells differ in the secretion of cytokines such as IL-1β and IL-17 and are also found in cellular infiltrates in renal biopsies from patients with lupus nephritis (80). Similarly, CD4^+^ T cells that express the Th1-associated chemokine receptor CXCR3 are abundantly present in the inflamed kidneys and their numbers in the urine are predictive for disease flares (81). More recently T follicular helper cells (Tfh) have been recognized as an important T cell population in SLE. Tfh cells are a heterogenous subset of CD4^+^ T cells that have the capability to migrate into the lymphoid follicles *via* the chemokine receptor CXCR5 and to induce the activation and differentiation of autoreactive B cell as part of the germinal-center reaction (4). In peripheral blood of patients with SLE numbers of Tfh cells, in particular of so-called circulating precursor Tfh cells, are elevated and correlate with disease activity (5, 82). CD4^+^ T cells lacking expression of the co-stimulatory receptor CD28 (CD4^+^CD28^lo^ cells), which are considered to represent chronically activated memory/effector CD4^+^ T cells, where shown to be expanded and to produce IFN-γ in patients with moderately active SLE (83). In patients with juvenile-onset SLE, elevated CD8^+^ effector memory T-cell frequencies indicated more persistently active disease over time. Active SLE is further characterized by a decline in CD4^+^FoxP3^+^CD127^lo^ Treg that express high levels of CD25 (CD25^hi^ Treg), a subset of Treg with a high suppressive capacity, and by an imbalanced proliferation between Treg and Tcon in favor of an enhanced Tcon proliferation (17). The reduced frequencies of CD25^hi^ Treg and the Treg/Tcon proliferation imbalance, which both correlated with disease activity, are typical indicators of a low availability of IL-2 and constitute the most relevant defects in Treg biology in SLE, which, however, can be corrected by treatment with low doses of IL-2 (17, 19, 21). Nevertheless, although the central role of T cells in established SLE has been well recognized over the last decades, phenotypic alterations of immune cells in a pre-diseased state still remain poorly explored due to lack of material from humans.

## Abnormal T cell phenotype in early murine SLE

Similar to other autoimmune diseases, it is proving difficult to study the origin and development of SLE before the onset of clinical symptoms in humans, and hence, mouse models of SLE provide valuable tools for the assessment of alterations at a cellular and molecular level before disease onset and during the progression of disease.

The NZBW mouse model is considered to authentically resemble most features of human SLE (35, 36). These mice spontaneously develop the lupus-like disease within 4 to 6 months of age, which provides a condition to study cellular and molecular changes of immune cells at the pre-diseased stage before disease onset. It has long been shown that immune responses such as the balance between T cell populations are altered by age in NZBW mice (84, 85). More recent studies indicated that increased CD4^+^ T cell activation and memory differentiation are detectable in lymphoid organs a long time prior to the appearance of clinical manifestations and even before relevant titers of the autoantibodies can be measured in the plasma, suggesting that aberrant T cell activation is an early event in this autoimmune condition (16). We have investigated the phenotypic changes of conventional CD4^+^FoxP3^-^ T cells (Tcon) and CD4^+^FoxP3^+^ Treg during disease progression in NZBW mice including young clinically healthy mice as well mice at the disease onset and with established disease (16). CD4^+^ Tcon from lymphoid organs of young, clinically healthy mice at an age between 8-12 weeks already showed signs of increased activation and memory formation evidenced by higher frequencies of CD69^+^ and CD44^+^ among CD4^+^ Tcon compared to healthy BALB/c mice. Frequencies and numbers of activated and memory CD4^+^ Tcon and IFN-γ producing Th1 cells further increased substantially during progression to disease onset and active disease. A lower prevalence of CD4^+^FoxP3^+^ Treg, which had an intact suppressive function, was already detectable in young pre-diseased NZBW mice compared to BALB/c mice. In parallel, these mice also had higher frequencies of CD69^+^ and CD44^+^ Treg suggesting that Treg activation with the attempt to counteract the increased Tcon activation occurs already early in disease development. Phenotypically, Treg from young mice still expressed normal levels of CD25 and IL-2 production by CD4^+^ T cells was also not impaired in young mice indicating that, in contrast to the later disease stages, lack of IL-2 may not be responsible for the low prevalence of Treg which instead is rather genetically determined (16). This is supported by studies in congenic mouse strains related to the NZBW strain that indicated that the low prevalence of Treg was linked to the disease-related Sle1a locus (86). Alternatively, the Treg deficiency may be caused by an impaired thymic Treg generation, however, we found that the numbers and proliferation rates of thymic Treg were normal in young NZBW mice (16). A decrease in CD25 expression in Treg and a diminished proliferation ratio between Treg and Tcon, which are indicators of Treg exhaustion due to IL-2 deficiency, could be observed earliest at the onset stage of disease, when IL-2 production by CD4^+^ T cells was also found to be significantly impaired (16), indicating that IL-2 deficiency is an acquired and potentially reversible phenomenon in SLE. Although the origins of IL-2 deficiency are certainly complex and not fully understood, we propose that it might be caused by the repression of IL-2 synthesis that occurs in chronically activated Tcon (21). Similar phenotypic alterations of Treg and Tcon could also be observed in the autoimmune susceptible NZB parental strain that develops a milder form of lupus, and to a lesser extent also in the clinically healthy NZW strain, suggesting that genetic alterations from both strains contribute to T cell hyperactivity in murine lupus (16).

## Abnormal T cell signaling and metabolism in early murine SLE

As described previously in humans, murine SLE T cells also exhibit rewiring of their TCR, in which expression of the CD3ζ chain is reduced (45). This reduction or even complete deletion leads to a severe systemic inflammatory response in mice (87). However, the pathologic changes in T cell signaling are not limited to this single signaling pathway. Interestingly, the entire CD4^+^ T cell life span from activation and proliferation to differentiation is strictly regulated by cellular metabolism (88, 89). A recent study in lupus-prone *B6.Sle123* and in SLE patients demonstrated that both aerobic glycolysis and mitochondrial oxidative phosphorylation are elevated in CD4^+^ T cells (90). The pathophysiological relevance of these findings was confirmed by showing that the application of the glycolysis inhibitor 2-deoxy-D-glucose and of the mitochondrial metabolism inhibitor metformin, were capable to suppress autoimmunity, decrease IFN-γ and IL-17 production and restore IL-2 synthesis, indicating that an altered cellular metabolism contributes to chronic T cell activation in SLE (90, 91). These data were collected at the onset stage of disease; however, it is reasonable to assume that corresponding events also occur at the pre-diseased stage of SLE and serve as initiators for subsequent pathological changes in cellular metabolism. In addition, and similar to studies in humans, the mTOR inhibitor rapamycin was capable to restore T cell metabolism and to decrease disease activity in lupus-prone MRL/lpr mice (92, 93).

## Proposed model of T cell dysregulation in SLE pathogenesis

**Figure 1:**
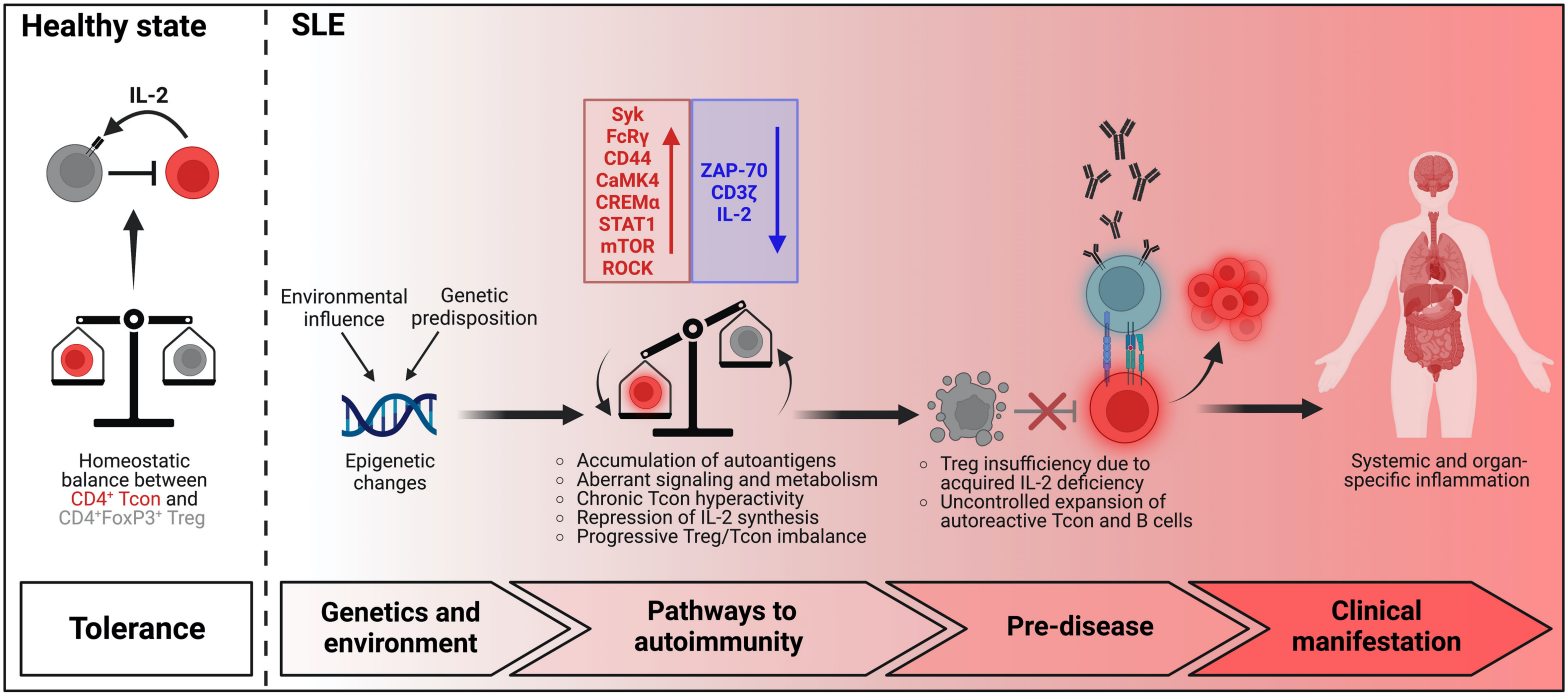
Model of T cell dysregulation in SLE pathogenesis.

Taking current knowledge into consideration, we propose a simplified model that may explain the gradual and progressive failure to adequately regulate the activation of autoreactive T cells in the immune pathogenesis of SLE which facilitates chronic activation of pathogenic T cells and promotes the generation of a robust autoimmune memory (**Figure 1**). In health there is a homeostatic balance between Treg and autoreactive Tcon that prevents the development of autoimmunity. In SLE distinct genetic alterations modified by environmental factors contribute to disease pathology. Initially, the accumulation of nuclear autoantigens which also serve as endogenous danger signals and induce the expression of pro-inflammatory cytokines, in particular of type-1 interferons, leads to the presentation of autoantigens by dendritic cells in an inflammatory context to autoreactive Tcon in the lymphoid tissues and consecutively to their activation and differentiation into effector/memory T cells and Tfh cells. In the early stage, the expansion of autoreactive T cell clones, which is facilitated by aberrant signaling and metabolism causing a lowered activation threshold, is partially counter-regulated by a functionally intact, yet already numerically restricted Treg population. The presence of pro-inflammatory cytokines upon antigen recognition also confers resistance in Tcon to Treg mediated suppression which further enhances the escape of autoreactive Tcon from immune regulation. During further progression of disease and due to the persistence of autoantigens and inflammatory signals, the pool of autoreactive effector/memory Tcon continuously expands, while their chronic and repetitive activation leads to the repression of IL-2 synthesis. The decreasing availability of IL-2 in turn impairs the adequate expansion of the Treg population in order to sufficiently counter-regulate the hyperactivity of Tcon which further facilitates the escape of autoreactive T cells and the chronicity of pathogenic T cell responses. This vicious cycle of a self-amplifying disruption of Treg homeostasis leading to progressive and chronic hyperactivity of autoreactive Tcon may continue for several years until numbers of Treg decline below a critical size and clinical manifestations emerge.

## Perspective

While the role of T cells and their dysregulation in established SLE is currently relatively well understood, little is still known about the mechanisms and molecular pathways which drive the escape from immune regulation and the chronicity of pathogenic T cell responses in the very early stage of disease. Continuing research efforts in this field provide the unique opportunity to identify novel therapeutic targets that might be capable to prevent the occurrence of clinical manifestations or to induce long-lasting remission.

## Author contributions

All authors listed have made a substantial, direct, and intellectual contribution to the work and approved it for publication.

## Funding

German Research Foundation, GRK2633.

## Conflict of interest

The authors declare that the research was conducted in the absence of any commercial or financial relationships that could be construed as a potential conflict of interest.

## Publisher’s note

All claims expressed in this article are solely those of the authors and do not necessarily represent those of their affiliated organizations, or those of the publisher, the editors and the reviewers. Any product that may be evaluated in this article, or claim that may be made by its manufacturer, is not guaranteed or endorsed by the publisher.
